# Assessment of Clinical Complete Response After Chemoradiation for Rectal Cancer with Digital Rectal Examination, Endoscopy, and MRI: Selection for Organ-Saving Treatment

**DOI:** 10.1245/s10434-015-4687-9

**Published:** 2015-07-22

**Authors:** Monique Maas, Doenja M. J. Lambregts, Patty J. Nelemans, Luc A. Heijnen, Milou H. Martens, Jeroen W. A. Leijtens, Meindert Sosef, Karel W. E. Hulsewé, Christiaan Hoff, Stephanie O. Breukink, Laurents Stassen, Regina G. H. Beets-Tan, Geerard L. Beets

**Affiliations:** Department of Radiology, Maastricht University Medical Centre, Maastricht, The Netherlands; Department of Epidemiology, Maastricht University Medical Centre, Maastricht, The Netherlands; Department of Surgery, Maastricht University Medical Centre, Maastricht, The Netherlands; Department of Surgery, Laurentius Hospital Roermond, Roermond, The Netherlands; Department of Surgery, Atrium Medical Centre, Heerlen, The Netherlands; Department of Surgery, Orbis Medical Centre, Sittard, The Netherlands; Department of Surgery, Leeuwarden Medical Centre, Leeuwarden, The Netherlands

## Abstract

**Background:**

The response to chemoradiotherapy (CRT) for rectal cancer can be assessed by clinical examination, consisting of digital rectal examination (DRE) and endoscopy, and by MRI. A high accuracy is required to select complete response (CR) for organ-preserving treatment. The aim of this study was to evaluate the value of clinical examination (endoscopy with or without biopsy and DRE), T2W-MRI, and diffusion-weighted MRI (DWI) for the detection of CR after CRT.

**Methods:**

This prospective cohort study in a university hospital recruited 50 patients who underwent clinical assessment (DRE, endoscopy with or without biopsy), T2W-MRI, and DWI at 6–8 weeks after CRT. Confidence levels were used to score the likelihood of CR. The reference standard was histopathology or recurrence-free interval of >12 months in cases of wait-and-see approaches. Diagnostic performance was calculated by area under the receiver operator characteristics curve, with corresponding sensitivities and specificities. Strategies were assessed and compared by use of likelihood ratios.

**Results:**

Seventeen (34 %) of 50 patients had a CR. Areas under the curve were 0.88 (0.78–1.00) for clinical assessment and 0.79 (0.66–0.92) for T2W-MRI and DWI. Combining the modalities led to a posttest probability for predicting a CR of 98 %. Conversely, when all modalities indicated residual tumor, 15 % of patients still experienced CR.

**Conclusions:**

Clinical assessment after CRT is the single most accurate modality for identification of CR after CRT. Addition of MRI with DWI further improves the diagnostic performance, and the combination can be recommended as the optimal strategy for a safe and accurate selection of CR after CRT.

In approximately 15–25 % of patients with rectal cancer who are treated with chemoradiotherapy (CRT), no residual tumor is found in the resection specimen, indicating a pathologic complete response (CR; ypT0N0).^1^ The increasing interest in organ-saving treatment through local excision or even a nonoperative treatment (a watch-and-wait strategy) demands a reliable method to identify patients with CR.^2^,^3^ Digital rectal examination (DRE) and endoscopy have been the main assessment tools to evaluate the response when the aim was to avoid surgery in specific indications, such as after contact radiotherapy in small rectal cancers.^4^ In the studies by Habr-Gama et al., who explored nonoperative treatment for CR in a wider group of patients, DRE and endoscopy also served as main selection tools.^2^,^5^ A drawback of endoscopy is that it only provides information on the luminal side and not on the deeper layers and the mesorectum. MRI can provide this additional information, which can be critical for decision making.^6^ Although MRI has been widely adopted for the primary staging of rectal cancer, restaging after CRT with standard T2-weighted (T2W) MRI is hampered by the difficulty of distinguishing fibrosis from viable tumor, often leading to incorrectly classifying fibrosis as residual tumor.^6^–^8^

Recently, diffusion-weighted MRI (DWI) has been shown to provide more accuracy than T2W-MRI.^9^ Initially in our center we relied on MRI as the first restaging method and used endoscopy for further evaluation when MRI was suggestive of a CR.^3^,^10^ With this selection strategy, a substantial part of those with CR was missed. Therefore, we changed the restaging strategy to routinely include DRE and endoscopy in all patients.

The aim of this study was to evaluate the respective value of clinical examination, consisting of DRE and endoscopy, with T2W-MRI and DWI for the detection of CR after CRT.

## Methods

### Patients

Fifty consecutive patients were prospectively included within 3 years in a study on disease restaging after CRT. Patients provided written informed consent for this restaging study. CRT was indicated for a (1) very distal tumor or (2) T4 tumor or (3) T3 tumor with involved mesorectal fascia and/or N1 disease with distal or midrectal location or (4) N2 status. CRT consisted of 28 fractions of 1.8 Gy radiation with capecitabine 825 mg/m^2^. Restaging was scheduled 6–8 weeks after completion of CRT.

### Clinical Assessment: DRE and Endoscopy

**Fig. 1:**
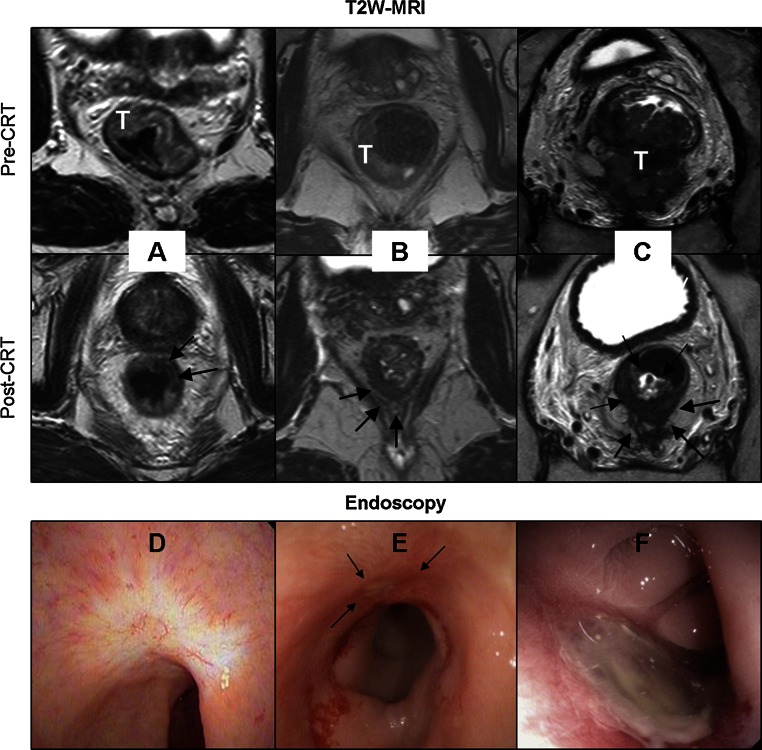
Response assessment with T2W-MRI (**a**–**c**) and with endoscopy (**d**–**f**). Pre- and post-CRT MR images are shown. T indicates tumor; arrows indicate scar or residual tumor after CRT. **a** Typical CR at T2W-MRI, **b** equivocal image at T2W-MRI, and **c** obvious residual tumor at T2W-MRI. **d** Typical endoluminal image of CR with white scar with teleangiectasia. **e** Small ulcer with smooth edges (*arrows*) but without residual polypoid tissue. Patients imaged in (**d**) and (**e**) experienced sustained clinical CR at follow-up. **f** Example of large ulcer that was deemed residual tumor after CRT

The patients were examined by one of three colorectal surgeons (GB, SB, LS). At DRE, findings were classified as: (1) normal bowel wall, (2) subtle residual abnormality of the bowel wall, and (3) obvious residual tumor. All patients underwent flexible endoscopy (Pentax Medical Netherlands, Uithoorn, The Netherlands) of the rectum after a rectal phosphate enema. Only white light imaging was used with HDTV, and the images of the tumor area were digitally stored. CR was defined as the absence of residual tumor with only a flat, white scar with or without teleangiectasia (Fig. 1). A small, flat ulcer with smooth edges without signs of residual polypoid tissue was considered to be a potential CR (Fig. 1). Every other type of ulcer or mass was considered as definite residual tumor (Fig. 1). A biopsy was only performed in equivocal cases, as judged by the surgeon during the endoscopy. Biopsy results that indicated tumor or high-grade dysplasia were considered proof of residual tumor. Absence of tumor or high-grade dysplasia in biopsy samples was not considered definite proof of CR because of the risk of sampling error. For the purpose of this study, two experienced clinicians (GB and MM, blinded to the MRI results and further clinical outcome), in consensus, rated the combination of the DRE and endoscopy findings and assigned a confidence level score for the overall clinical assessment (Table 1).

**Table 1:** Definitions of confidence level scores for assessment of complete response for every modality

CL	Clinical assessment	T2W-MRI findings	DWI findings
CL 0	Positive biopsy result or gross residual tumor at endoscopy with or without palpable mass at DRE	Gross residual isointense mass and/or involved nodes	Marked hyperintense signal at former tumor location on b1000 images with low ADC
CL 1	Visible (with or without palpable) mass or polypoid tissue with negative biopsy	Small residual isointense mass and/or involved nodes	Small but obvious area of hyperintense signal at former tumor location on b1000 images with low ADC
CL 2	Ulcer with irregular borders and small palpable ridge, ulcer or wall thickening with negative biopsy	Irregular wall thickening with both hypointense and isointense signal	Possible foci of hyperintense signal on b1000 images at former tumor location with low ADC in an area of irregular wall thickening
CL 3	Small nonpalpable ulcer with regular borders and negative biopsy	Pronounced hypointense wall thickening without isointense signal and no involved nodes	No clear areas of residual hyperintense signal on b1000 images at former tumor location
CL 4	White scar with teleangiectasia, no palpable lesions and negative biopsy	Normalized rectal wall or only subtle wall hypointense wall thickening and no involved nodes	No residual hyperintense signal on b1000 images or low ADC at former tumor location

*CL* confidence level, *T2W*-*MRI* T2-weighted MRI, *DWI* diffusion-weighted imaging, *DRE* digital rectal examination, *ADC* apparent diffusion coefficient

### MRI

**Fig. 2:**
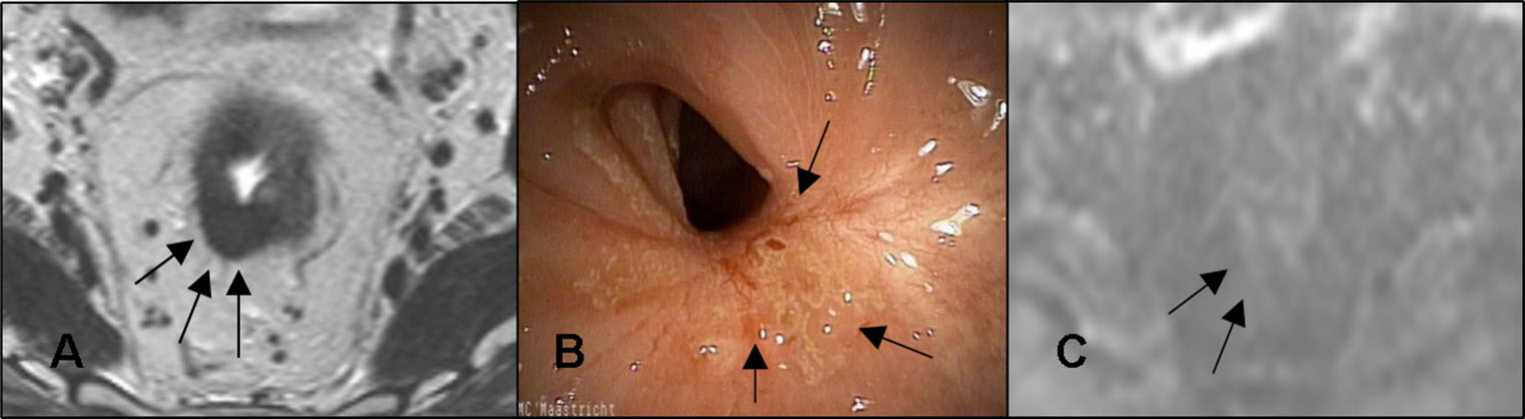
Example of patient with a CR where T2W-MRI (**a**) revealed marked hypointense residual wall thickening resulting with an equivocal (confidence level 2) score. Clinical assessment (**b**) revealed a white scar with some stenosis and distortion, and small superficial ulceration, also resulting in an equivocal score. DWI (**c**) revealed absence of diffusion restriction indicating CR

All MRI examinations were performed at 1.5 T using a phased array body coil [Intera (Achieva) or Ingenia, Philips Medical Systems, Best, The Netherlands] and included T2W-MRI in three orthogonal directions (axial, sagittal, and coronal). Additional axial diffusion-weighted images were obtained with b0 as the lowest and b1000 as the highest b value. The sequence details are shown in Appendix. An intravenous bolus injection of 20 mg of butylscopolamine (Buscopan; Boehringer Ingelheim, Ingelheim, Germany) was administered to reduce peristaltic movement; patients did not receive bowel preparation. An apparent diffusion coefficient (ADC) map was automatically calculated. The T2W-MRI and DWI axial images were angled in identical planes. A reader with 5-year specific experience in rectal cancer MRI (DL) scored the T2W-MRI images together with the DWI (b1000 and ADC) images for the presence of CR with confidence level scores (Table 1). ycN0 was assessed on the basis of size and morphology criteria.^11^ The reader had the pre-CRT MRI at her disposal and was blinded to the endoscopy results and histopathology (if available). Figure 1 shows examples of a CR, equivocal score, and obvious residual tumor by T2W-MRI. Figure 2 illustrates an example of DWI being decisive in determining a CR when clinical assessment and T2W-MRI show equivocal results.

### Reference Standards

Histopathology of the total mesorectal excision (TME) resection specimen was used as the reference standard, with both high-grade dysplasia and carcinoma considered as residual tumor. CR was defined as ypT0N0. Surgical specimens were evaluated according to the method of Quirke and Dixon.^12^ Some Patients underwent clinical exams and endoscopy + DWI-MRI in the first year of follow-up every 3 months and from the second year this was performed every 6 months.^3^ For these patients, a local recurrence-free follow-up time of ≥12 months was used as a surrogate end point for a CR. For patients who underwent a local excision of the remaining scar [transanal endoscopic microsurgery (TEM)], the reference standard consisted of histopathology of the specimen with ≥12 months’ follow-up by MRI and endoscopy.

### Statistical Analysis

Statistical analyses were performed with SPSS Statistics 20 (IBM, Armonk, NY) and Stata 11.0 (StataCorp, College Station, TX). Receiver operator characteristics (ROC) curves were constructed with confidence levels to assess the diagnostic performance of clinical assessment and MRI. The areas under the ROC curve (AUC) with corresponding sensitivities and specificities were calculated for all modalities. The cutoff for sensitivity and specificity was set between confidence level 2 and 3 at the start of the study for both clinical assessment and MRI. AUCs were compared between modalities by the method of Hanley and McNeil.^13^ With logistic regression analyses, predicted probabilities were calculated for the diagnostic performance of the combination of MRI with clinical assessment. With these predicted probabilities, a ROC curve was constructed. The positive outcome measure was the presence of a CR. In addition to the diagnostic performance of the modalities, the positive and negative likelihood ratios were calculated for the following: (1) clinical assessment, (2) T2W-MRI with DWI, and (3) both modalities combined.^14^ These likelihood ratios were used to calculate posttest probabilities for a CR when the modalities are combined by the multiplying pretest odds with the likelihood ratios. *P* values of <0.05 were considered statistically significant.

## Results

### Patients

Of the 50 included patients, 33 were men (66 %). The median age was 67.5 years (range 34–88 years). Thirty-four patients underwent a TME, and six underwent a TEM as part of a study.

Seventeen patients experienced a CR (34 %): eight after surgery (two after TEM, six after TME), and nine had a clinical CR and were followed with a watch-and-wait policy, with a median follow-up of 17 months (range 12–20 months). One patient with residual tumor had ypT0N1 disease. At primary staging, 72 % of patients had a cT3 tumor (36 of 50), 20 % (10 of 50) had a cT2 tumor, and 8 % (4 of 50) had a cT4 tumor. At primary presentation, 38 (76 %) of 50 tumors were palpable at DRE. The median interval between the last radiation dose and the restaging was 8 weeks (range 3–35 weeks), and between restaging MRI and histopathology 9.5 days (range 0–74 days). The median time between clinical assessment and restaging MRI was 0 days (range 0–56 days). At endoscopy, biopsies were performed in 29 patients; findings were benign in 20 patients, eight of which turned out to be false CRs after surgery. In three patients the biopsy results revealed adenocarcinoma, and high-grade dysplasia was found in six. In this small sample, the sensitivity of a biopsy for persistent tumor was 9 (53 %) of 17, and the negative predictive value for persistent tumor was 12 (60 %) of 20.

### Diagnostic Performance

**Fig. 3:**
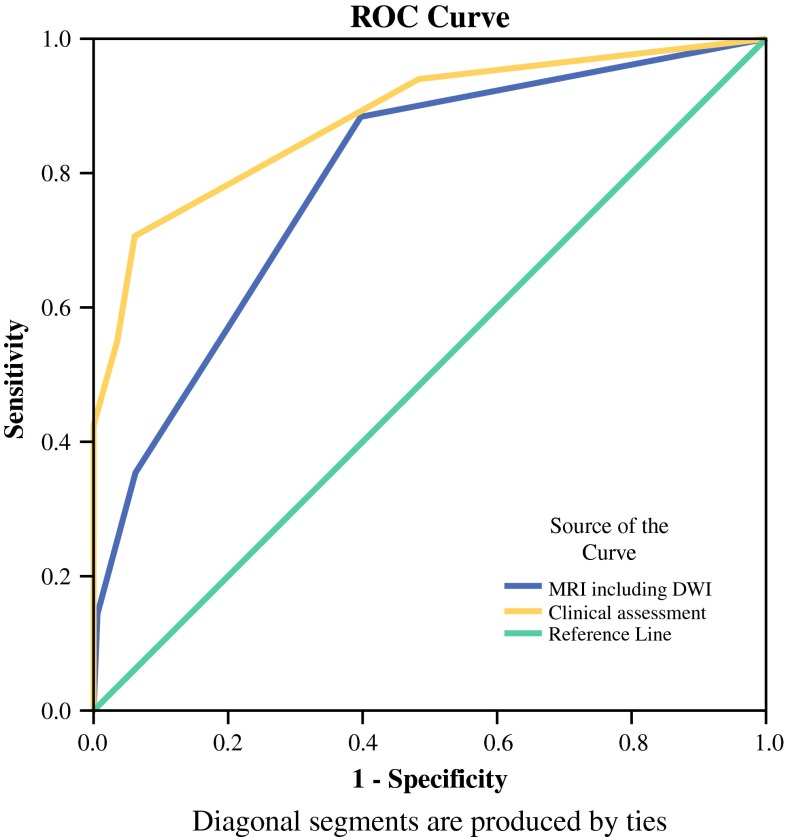
ROC curves for modalities. Clinical assessment consists of endoscopy, DRE, and biopsy result (if available)

Figure 3 shows the ROC curves for MRI and clinical assessment. Table 2 shows the diagnostic parameters for the modalities. For clinical assessment, the AUC was 0.88 (95 % confidence interval 0.78–0.99), and sensitivity and specificity were 53 and 97 %, respectively. For T2W-MRI and DWI, the AUC was 0.79 (95 % confidence interval 0.66–0.92), with a sensitivity of 35 % and a specificity of 94 %. The difference between clinical assessment and T2W-MRI and DWI was not statistically significant (*P* = 0.17).

**Table 2:** Diagnostic parameters for clinical assessment, T2W-MRI and DWI, and all assessment modalities

Parameter	Clinical assessment	T2W-MRI and DWI	All
Sensitivity	53 %	35 %	71 %
Specificity	97 %	94 %	97 %
PPV	90 %	75 %	NA
NPV	80 %	74 %	NA
AUC	0.88 (0.78–0.99)	0.79 (0.66–0.92)	0.89 (0.79–0.99)
LR positive	17.67	5.83	–
LR negative	0.48	0.69	–
Positive posttest probability	90 %	75 %	98 %
Negative posttest probability	20 %	26 %	15 %

Positive posttest probability is the probability of CR when both tests have positive results (indicate CR) and negative posttest probability is the probability of CR when both tests have negative results (indicate residual tumor). Diagnostic parameters were calculated on the basis of predefined cutoff in confidence levels between 2 and 3

*T2W*-*MRI* T2-weighted MRI, *DWI* diffusion-weighted MRI, *NA* not applicable, *PPV* positive predictive value, *NPV* negative predictive value, *AUC* area under the receiver operator characteristic curve, *LR* likelihood ratio

### Probability for CR with Combination of Methods

The positive likelihood ratio for a CR for clinical assessment was 17.67 and for T2W-MRI and DWI 5.83. The posttest probability (calculated with the positive likelihood ratios) for the presence of a CR for clinical assessment was 90 % and for T2W-MRI and DWI MRI was 75 %. When all three modalities were combined, the posttest probability for a CR was 98 %, indicating that when all three modalities predict a CR, this is correct in 98 % of the cases, with only a 2 % risk of missing residual tumor. The negative likelihood ratio was 0.48 for clinical assessment and 0.69 for T2W-MRI with DWI. These likelihood ratios led to a posttest probability of a CR of 20 % for clinical assessment and 26 % for T2W-MRI and DWI when either of the modalities indicates residual tumor. When combining all modalities, this decreases to 15 %, meaning that when all three modalities indicate residual tumor, there still is a 15 % chance for a CR.

**Fig. 4:**
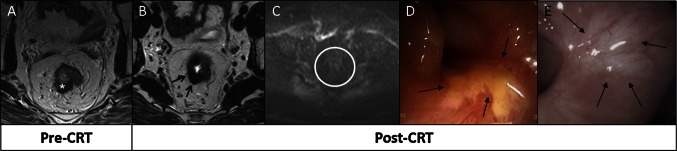
**a** Tumor (*asterisks*) before CRT. After CRT at T2W-MRI (**b**), fibrosis (*arrows*) is found with absence of high signal on DWI (**c**), suggestive of a CR. At endoscopy (**d**), a residual ulcer (*arrows*) is found, indicating residual tumor. Patient refused surgery and has been followed up for 3.5 years with stable MR image and a healed ulcer (**e**, *arrows*), so is classified as having experienced CR

**Fig. 5:**
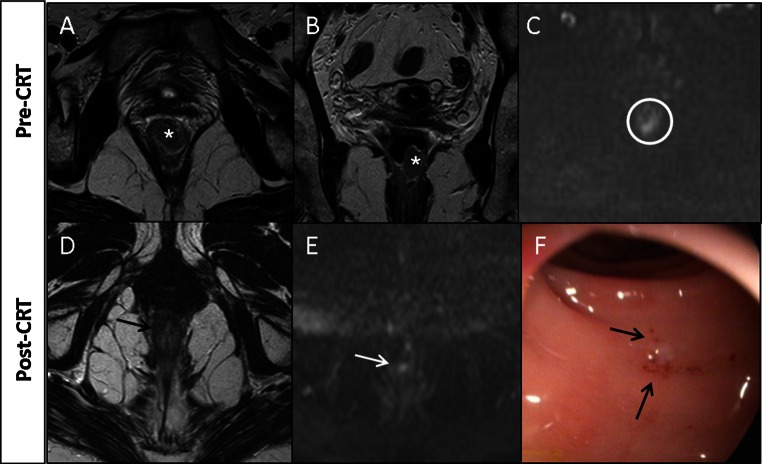
**a, b** Distal tumor (*asterisks*) before CRT at T2W-MRI and **c** DWI. After CRT at T2W-MRI (**d**) and DWI (**e**), residual tumor was suspected (*arrows*). At endoscopy (**f**), CR (*arrows*) was determined, and the patient was treated with wait-and-see policy. After 3 months, DWI became normal; patient remained free of recurrent disease at 3.8 years of follow-up

Figures 4 and 5 show how the modalities complement each other in assessment of response after CRT.

## Discussion

In this study, clinical assessment including DRE and endoscopy proved to be the most accurate strategy to select patients who may experience CR. The addition of MRI to DWI, however, increases the identification CR rate to a level that is reliable for clinical decision making. When clinical assessment, T2W-MRI, and DWI all indicate a CR, this is correct in 98 % of the cases, missing residual tumor in only 2 %. When all modalities indicate residual tumor, in 15 % of the cases, there is actually is a CR.

Rigid endoscopy and DRE have been the standard of response assessment in the past treatment of rectal cancer with radiotherapy alone.^15^ A continuing decrease in size and disappearance of the tumor with healing of the mucosa were generally considered signs of a clinical CR. In later series, rigid endoscopy was often replaced by flexible sigmoidoscopy and imaging with endorectal ultrasound with or without MRI was added.^16^ Habr-Gama et al. showed that whitening of the mucosa (with or without teleangiectasia) or a complete normalization of the tumor bed should be considered a CR, a finding that is confirmed in the current study.^5^ The literature has shown that residual tumor can be found in any layer of the bowel wall, regardless of tumor stage or presence of ulcer.^17^–^20^ Therefore, a major concern of clinical and endoscopic assessment of response is the risk of missing such scattered tumor deposits, leading to the cautious strategy to perform a major resection whenever potential residual tumor is suspected. This approach and the degree of subjectivity of clinical assessment are illustrated in a study where DRE only detected 3 of 14 patients with a CR, while a CR was never falsely predicted in the 80 patients with residual tumor.^21^ Given the fact that sampling errors occur regularly in case of residual tumor, biopsies have only limited clinical value for ruling out residual cancer.^22^ This variability of tumor scatter could explain the 10–30 % early and late regrowths in series of watchful waiting and underscores the need for imaging methods that evaluate the deeper layers of the bowel wall and the mesorectum.^3^,^23^–^25^

Endorectal ultrasound, FGD-PET-CT, and T2W-MRI all have shown insufficient diagnostic performance to detect residual tumor in fibrosis after CRT, and the strategy to err on the safe side leads to overestimation of residual tumor.^9^,^26^–^28^ The accuracy of T2W-MRI can be improved by adding a DWI sequence, generating qualitative and quantitative information on the cellular architecture on the basis of differences in movement (diffusion) of water protons within the various tissues. Malignant tissues, with a high cellular density, show restricted proton movement leading to an increased signal. A meta-analysis on response assessment in rectal cancer has shown that DWI improves the diagnostic performance, mainly through increasing the detection rate of response up to 84 %, along with a very low risk of missing residual tumor.^9^ In the present study, combined prediction of a CR on clinical assessment as well as MRI including DWI resulted in a very high predictive value for a CR of 98 %. With this strategy, however, about one in three CRs is missed.

A clinically relevant question is whether it is necessary to err so much on the safe side. A transanal excision of the scar can provide histologic proof when there is an equivocal clinical and radiologic picture. The disadvantage is that follow-up is somewhat more difficult, and in the event of a recurrence, the ideal surgical plane may have been violated. Another alternative is to extend the observation interval for an additional 1–2 months, as it can take several months before the full effect of the CRT becomes evident.^29^ The two approaches of local excision and extending the observation interval will increase the number of patients who can be offered organ preservation.

The most practical and cost-efficient strategy to identify patients likely to experience clinical CR also depends on local logistics and expertise. Currently, experience with clinical assessment after CRT is limited and lacks standardization. Additionally, clinical assessment has a high degree of observer variability. When restaging MRI is part of the routine, it could serve as a first selection tool and avoid unnecessary endoscopies in patients with obvious residual tumor. When restaging MRI is not part of the routine, DRE is by far the most cost-efficient way to determine gross residual tumor. Regardless of the first screening method, it is prudent in patients considered candidates for organ preservation to use all methods: DRE, endoscopy, and MRI. MRI provides information on the presence of tumor in the deeper layers of the rectal wall, the mesorectum, and the lymph nodes, and it provides detailed images that can be used for serial follow-up.^10^

The most important limitation of the present study is the relatively small sample size, and thus some caution in the interpretation of the results is required. Second, the prevalence of CR after CRT (34 %) is higher than generally reported in the literature (15–25 %) as a result of the referral pattern to our center of patients with a good response. Another limitation is that in some patients, the reference standard was a lasting clinical CR at follow-up of at least 1 year, with a median of 16.5 months. Although most regrowths occur within the first year of follow-up, it cannot be excluded that some will occur later. Additionally, the range in interval between last radiation dose and response evaluation and surgery is wide, which could have an influence on our results.

In conclusion, clinical assessment with DRE and endoscopy is the most accurate strategy to identify patients likely to experience CR, and it should be incorporated in a post-CRT restaging strategy when organ preservation is considered. Addition of MRI (including DWI) further improves diagnostic performance, and the combination of the two can be recommended as a strategy for a safe and accurate selection of CR after CRT.

## Appendix

The standard rectal imaging protocol consisted of T2W fast-spin echo sequences in three orthogonal planes (TR 8456–9558 ms, TE 130 ms, 25 echotrain length, 2–6 NSA, 0.78 × 1.14 × 3.00 mm voxel size, 30 slices, 4.37–6.03 min acquisition time). Diffusion-weighted imaging (TR/TE 4829/70 ms, EPI factor 53–61, 5 NSA, 1.8 × 2.3 × 5 mm acquisition voxel size, 24–50 slices, 5.33–10.37 min’ acquisition time) was performed with DWIBS in half of the patients and with SPIR/SPAIR in the remaining patients.
